# Glycine Cleavage System H Protein Is Essential for Embryonic Viability, Implying Additional Function Beyond the Glycine Cleavage System

**DOI:** 10.3389/fgene.2021.625120

**Published:** 2021-01-25

**Authors:** Kit-Yi Leung, Sandra C. P. De Castro, Gabriel L. Galea, Andrew J. Copp, Nicholas D. E. Greene

**Affiliations:** Great Ormond Street Institute of Child Health, University College London, London, United Kingdom

**Keywords:** glycine cleavage system, glycine cleavage system H protein, embryonic lethality, lipoylation, mouse models

## Abstract

Glycine cleavage system H protein (GCSH) is a component of the glycine cleavage system (GCS), a conserved protein complex that acts to decarboxylate glycine. Mutation of *AMT* or *GLDC*, encoding the GCS components aminomethyltransferase and glycine decarboxylase, can cause malformations of the developing CNS (neural tube defects (NTDs) and ventriculomegaly) as well as a post-natal life-limiting neurometabolic disorder, Non-Ketotic Hyperglycinemia. In contrast, it is unclear whether mutation of *GCSH* contributes to these conditions and we therefore investigated GCSH loss of function in mice. Mice that were heterozygous for a *Gcsh* null allele were viable and did not exhibit elevated plasma glycine. Moreover, heterozygous mutation of *Gcsh* did not increase the frequency of NTDs in *Gldc* mutant embryos. Homozygous *Gcsh* null mice were not recovered at post-natal stages. Analysis of litters at E8.5-10.5, revealed the presence of homozygous null embryos which were much smaller than littermates and had failed to develop beyond early post-implantation stages with no visible somites or head-folds. Hence, unlike null mutations of *Gldc* or *Amt*, which are compatible with embryonic survival despite the presence of NTDs, loss of *Gcsh* causes embryonic death prior to mid-gestation. Maternal supplementation with formate did not restore embryonic development beyond E7.5, suggesting that the primary cause of lethality was not loss of glycine cleavage activity or suppression of folate one-carbon metabolism. These findings suggest that GCSH has additional roles beyond function in the glycine cleavage system. We hypothesize that GCSH potentially acts in lipoylation of 2-oxoacid dehydrogenase proteins, as reported in bacteria.

## Introduction

**FIGURE 1:**
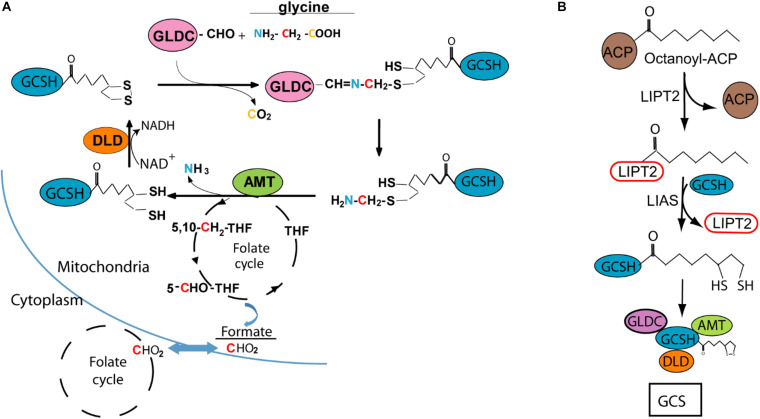
Glycine cleavage system H protein (GCSH) function and lipoylation. **(A)** Diagram of the glycine cleavage system showing decarboxylation of glycine by GLDC with transfer of the methylamine group to GCSH, followed by AMT mediated release of ammonia and transfer of the methylene group to tetrahydrofolate (THF), which enters the mitochondrial folate cycle that generates formate for transfer to the cytoplasm. **(B)** Lipoyl-GCSH is generated via LIPT2 (lipoyltransferase 2) acyl enzyme intermediate generated from octanoyl-ACP (acyl carrier protein), with subsequent transfer to GCSH and sulfur insertion by lipoic acid synthase (LIAS).

Glycine cleavage system H-protein (GCSH) is one of four enzymes which, together with glycine decarboxylase (GLDC), aminomethyltransferase (AMT), and dehydrolipamide dehydrogenase (DLD), make up the glycine cleavage system (GCS). This highly conserved protein complex is located at the mitochondrial membrane in eukaryotes and is the major route of glycine catabolism. GCS action involves oxidative cleavage of glycine with release of carbon dioxide (CO_2_) and ammonia (NH_3_) and transfer of a methylene group (–CH_2_–) to tetrahydrofolate, with concomitant reduction of NAD^+^ to NADH (Figure 1A; Kikuchi et al., 2008).

Based on analysis in the leaf of pea plants (*Pisum sativum*), the stoichiometry of the GCS is estimated at 4 GLDC :27 GCSH: 9 AMT :2 DLD (Oliver et al., 1990). GCSH plays a central role in the catalytic process, forming an amino-methyl intermediate with GLDC and acting as the acceptor of the methylamine group which is then transferred as the substrate for AMT (Figure 1A). This function of GCSH depends on modification by covalent addition of lipoic acid, as a cofactor, to form the active lipoyl-enzyme. The lipoyl moiety is believed to assembled on GCSH in a multi-step process, with addition of the octanoyl group followed by insertion of the sulphydryl groups (Figure 1B; Christensen et al., 2011; Cao et al., 2018b; Cronan, 2020). DLD mediates reduction of NAD^+^ and oxidation of the lipoyl group of GCSH (Figure 1A).

Loss of function of the GCS is predicted to cause accumulation of glycine and suppression of folate one-carbon metabolism (FOCM) and this is associated with post-natal neurometabolic disease and structural malformations of the developing brain including neural tube defects (NTDs) and ventriculomegaly. Accumulation of glycine in tissue and body fluids is a hallmark of Non-Ketotic Hyperglycinemia (NKH), a life-limiting inborn error of metabolism which presents in the neonatal period with hypotonia, apnea, and seizures. Subsequently, affected babies and children suffer complex epilepsy and profound developmental delay (Hoover-Fong et al., 2004; Swanson et al., 2015). Enlarged brain ventricles and/or hydrocephalus are also associated with NKH in a proportion of patients (Van Hove et al., 2000; Hennermann et al., 2012). Classic NKH, caused by mutation of GCS-encoding genes, is inherited as an autosomal recessive condition with the majority of patients carrying mutations in *GLDC* and around 20% carrying mutations in *AMT* (Kure et al., 2006; Kanno et al., 2007; Coughlin et al., 2017).

In addition to NKH, mutations in *GLDC* and *AMT* contribute to susceptibility to NTDs (Narisawa et al., 2012; Shah et al., 2016), severe birth defects which result from failed closure of the neural tube during embryonic development. To date, GCS mutations reported in NTDs are in heterozygous form suggesting that mutations in other genes and/or environmental factors also contribute to these cases, consistent with the multifactorial etiology of NTDs (Greene and Copp, 2014).

The potential role of *GLDC* and *AMT* mutations in some NTDs is supported by the occurrence of NTDs in mouse knockouts of the orthologous genes (Narisawa et al., 2012; Pai et al., 2015; Leung et al., 2017). Among *Gldc-*deficient mice that are not affected by NTDs, the occurrence of ventriculomegaly and hydrocephalus (resulting from stenosis of the aqueduct of Sylvius) similarly confirm that these are specific effects of GCS-encoding mutations in NKH patients (Autuori et al., 2017; Santos et al., 2020).

The loss of GCS activity in *Gldc-*deficient mice results in elevated glycine and a number of glycine derivatives in plasma and tissues, including liver and brain (Pai et al., 2015; Leung et al., 2020). In parallel, metabolic labeling shows that the contribution of glycine-derived one-carbon units to folate one-carbon metabolism is ablated (Leung et al., 2017) and the folate profile is perturbed, with a deficit of folates that carry one-carbon groups (Pai et al., 2015).

Whereas DLD acts in at least three other mitochondrial enzyme complexes, GLDC, AMT, and GCSH have been described as specific to the glycine cleavage system. However, unlike *GLDC* and *AMT*, mutations in *GCSH* have not been reported in patients with NTDs or NKH (Kure et al., 2006; Coughlin et al., 2017). However, a putative heterozygous mutation was identified in an individual with transient neonatal hyperglycinemia (Kure et al., 2002). In addition, patients have been identified in which elevated plasma glycine is associated with reduced GCSH activity and defects in addition of the lipoic acid moiety (Hiraga et al., 1981), with mutations identified in lipoic acid synthase, *LIAS* (Baker et al., 2014). In the current study, we investigated the effect of *Gcsh* loss of function in mice, in order to ask whether this revealed a potential role in NTDs and/or features of NKH.

## Materials and Methods

### Mice

Animal studies were carried out under regulations of the Animals (Scientific Procedures) Act 1986 of the United Kingdom Government, and in accordance with the guidance issued by the Medical Research Council, United Kingdom in *Responsibility in the Use of Animals for Medical Research* (July 1993). The *Gcsh^em1(IMPC)H^* (previously denoted H-GCSH-DEL653-EM1-B6N) mouse line was generated by CRISPR/Cas9 targeting at MRC Harwell, Mary Lyon Centre (Oxfordshire) on a C57BL/6N Tac background (Mianne et al., 2017). The *Gcsh* mutant allele (hereafter denoted *Gcsh*^–^) carried a deletion of 653 nucleotides (nucleotides 116,987,776–116,987,124) encompassing exon 3 of *Gcsh* (ENSMUSG00000034424).

### Genotyping

Genotyping of mice and embryos was carried out by PCR amplification of genomic DNA. Use of primers which flank the deletion (*Gcsh*_F2;5' -GCACGTAGTAGGGTTGACAAGT- 3' and Gcsh_R2; 5'-CGCGGATGGAGAACTGTAAGG-3') produces a PCR product of 1,033 bp from wild-type DNA whereas the deletion allele generates a smaller product of 380 bp. A confirmatory reaction (Gcsh_F2 primer and Gsch_R1; 5'- CAGGAAGCGTTGGGAGATGTTG-3') used a reverse primer which binds in the deleted region and therefore generated PCR product from wild-type but not Gcsh deleted DNA.

### Generation of *Gldc^–/–^*; *Gcsh^+/–^* Mice

*Gldc*-deficient mice, denoted *Gldc*^GT1^, carry a gene-trap construct in intron 2 of *Gldc* (Pai et al., 2015) and are maintained on a C57Bl/6J background. *Gldc^GT1/+^*; *Gcsh^+/^*^–^ males were generated by intercross of *Gcsh*^+/–^ males with *Gldc^GT1/+^* females. The double heterozygotes were used in timed matings with *Gldc^GT1/+^* females in order to generate embryos of genotype, *Gldc*^GT1/GT1^; *Gcsh^+/^*^–^.

### Collection of Embryos and Formate Supplementation

Timed matings were set up overnight and the day of finding a copulation plug was designated embryonic day 0.5 (E0.5). For embryo collection, the dam was killed by cervical dislocation and the uterus explanted into Dulbecco’s Modified Eagles Medium (DMEM) containing 10% fetal bovine serum, prior to dissection of embryos from the extra-embryonic membranes. In formate supplementation studies, the drinking water of pregnant dams was replaced on E0.5 with water containing 30 mg/ml sodium formate (Sigma) and mice were maintained on this water until litters were collected at E8.5 or E10.5 as in previous studies (Pai et al., 2015; Leung et al., 2017).

### Plasma Glycine Assay

Blood was collected by terminal cardiac exsanguination under isofluorane anesthetic, transferred to lithium-heparin tubes (BD Microtainer), immediately centrifuged at 3,000*g* for isolation of plasma and stored at −20°C. Glycine was labeled using the Kairos AccQ-tag derivation kit and analyzed on a Xevo ACQUITY UPLC I-Class PLUS System with Xevo TQ-S micro (Waters, United Kingdom). Derivatisation of glycine and UPLC-MS/MS conditions were in accordance with the manufacturer’s protocol.

### Confocal Microscopy Imaging of E7.5 Embryos

Confocal images of micro-dissected E7.5 DAPI-stained embryos were captured on a Zeiss Examiner LSM880 confocal microscope using a 20×/NA1.0 Plan Apochromat dipping objective. Images were also captured in reflection mode as previously described (Galea et al., 2017) and visualized as maximum projections or as 3D reconstructions in Fiji (Schindelin et al., 2012). For 3D reconstructions, reflection signal was enhanced using the local contrast enhancement tool (CLAHE) in-built in Fiji as previously described (Galea et al., 2018).

## Results

### The Deleted *Gcsh* Allele Results in Embryonic Lethality

The *Gcsh* gene consists of 5 exons which generate a 1,192 nucleotide transcript encoding a polypeptide of 173 amino acids that is processed to give a mature protein of 125 amino acids. We analyzed mice carrying a 643 base pair deletion that encompassed the entire exon 3 of *Gcsh*, creating a frame-shift after exon 2 (amino acid 73) and a predicted null allele (here denoted *Gcsh*^–^). *Gcsh^+/^*^–^ mice were viable and fertile and were indistinguishable from wild-type littermates. It has been reported that the electrocardiogram shows an abnormally long RR interval (time between R waves) in heterozygotes (*n* = 8 female, 7 male) compared with wild-type controls^1^.

No *Gcsh* homozygous mutant pups were recovered among the offspring of heterozygous matings, resulting in a clear distortion of the expected Mendelian ratio (*n* = 18 *Gcsh*^+/+^, 41 *Gcsh*^+/–^ and 0 *Gcsh*^–^*^/^*^–^). This was consistent with data subsequently reported from the MRC Harwell preweaning lethality screen and indicates lethality of homozygous *Gcsh* null mice at neonatal or pre-natal stage.

Loss of function of the GCS components *Gldc* and *Amt* also results in lethal phenotypes, but null embryos survive to late-fetal stages and exhibit neural tube defects (NTDs) with varying penetrance (Narisawa et al., 2012; Pai et al., 2015; Leung et al., 2017). Therefore, we asked whether *Gcsh*^–^*^/^*^–^ embryos were detected at pre-natal stages and if so whether NTDs were present. Experimental litters were generated by timed matings of *Gcsh*^+/–^ heterozygotes and litters collected at mid-gestation. Among an initial three litters collected at E10.5, a total of twenty embryos included only two conceptuses (10%) that genotyped as *Gcsh*^–^*^/^*^–^ (Table 1A). Moreover, in both cases the embryonic material consisted of only a small tissue fragment within the yolk sac without typical morphology of littermates. Hence, embryonic lethality occurs prior to E10.5, as further supported by the high rate of resorption in these litters which likely corresponds to *Gcsh*^–/–^ implantations (Table 1A).

**TABLE 1:** Frequency of embryo genotypes among litters from *Gcsh*^+/^*^–^* intercrosses.

A. *Gcsh^+/^^–^* × *Gcsh^+/^^–^* litters								
	Litters	Embryos (n)	Resorption (n)	Implantations litter	“Embryos” litter	Genotype		
						*Gcsh^+/+^*	*Gcsh^+/–^*	*Gcsh^–/–^*
E8.5	5	48	1	9.8	9.6	18	23	7*
						38%	48%	15%
E10.5	3	20	7	9.0	6.7	6	12	2*
						30%	60%	10%

**B. *Gcsh^+/^****^–^* **× *Gcsh^+/^****^–^* **litters with formate supplementation**								
	**Litters**	**Embryos (n)**	**Resorption (n)**	**Implantations litter**	**“Embryos” litter**	**Genotype**		
						***Gcsh*^+/+^**	***Gcsh^+/–^***	***Gcsh^–/–^***

E8.5-9.5	4	36	1	9.3	9.6	10	19	7^#^
						28%	53%	19%
E10.5	4	20	7	6.8	6.7	6	11	3^#^
						30%	55%	15%

*(A,B) Implantations were dissected and “embryos” (classified on the basis that some tissue was present inside an intact yolk sac) were genotyped. (A) Among untreated litters, the material that genotyped as Gcsh****^–/–^***(*) consisted of morphologically undefined tissue masses with no visible somites. (B) This was the case for the majority of Gcsh***^–/–^****embryos exposed to maternal formate supplementation (#), except 1/7 embryos at E9.5 had 2 somites (compared with 15 somites in littermates) while 1/7 embryos had headfolds but no somites. Similarly, 1/3 embryos at E10.5 was slightly more developed with 1 visible somite (in contrast to littermates that had 29 or more somites).*

**FIGURE 2:**
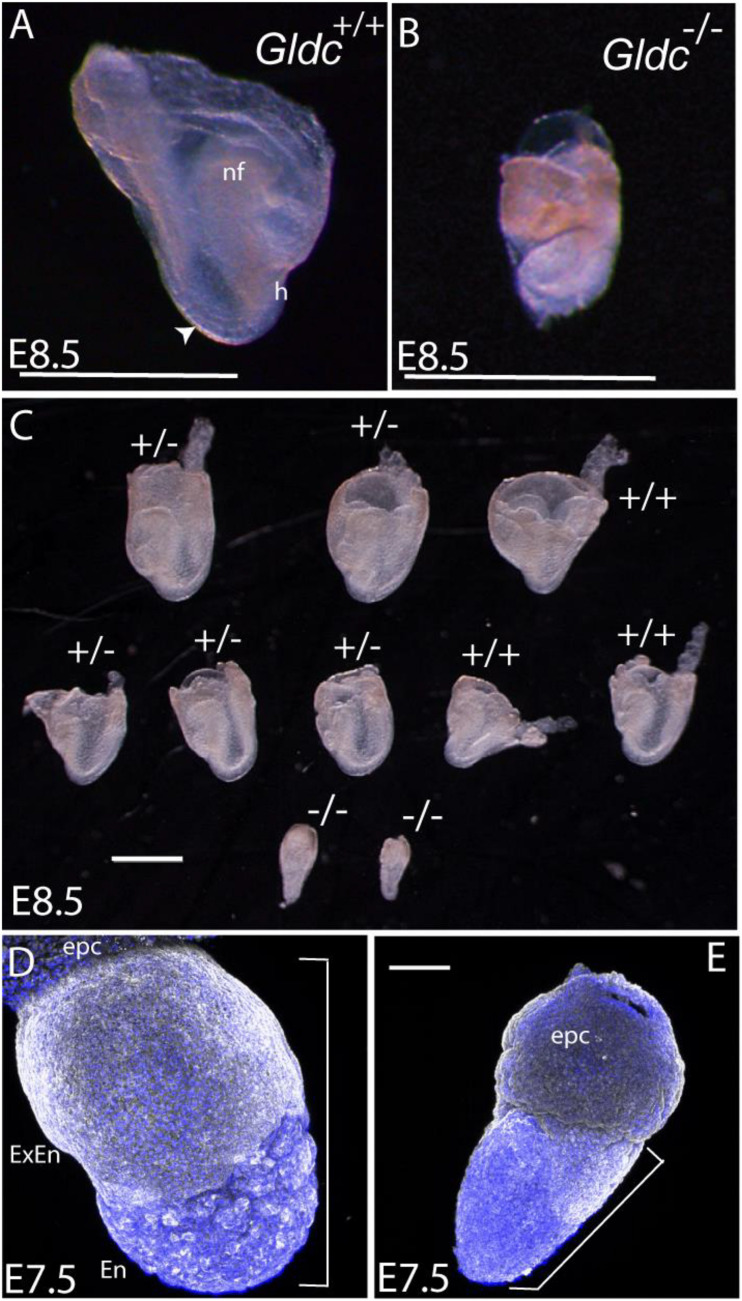
Morphological appearance of *Gcsh* null embryos. **(A,B)** Images at 8.5 show the typical appearance of **(A)** a wild-type embryo (inside the yolk sac), with visible somites (arrowhead), cranial neural folds (nf), and heart loop (h), whereas **(B)** a typical *Gcsh^–/–^* littermate is smaller and under-developed. **(C)** A representative litter of embryos (contained within the yolk sac) at E8.5, in which wild-type and *Gldc*^+/^*^–^* embryos had developed to the 4–5 somite stage, with clearly visible neural folds and heart structure. In contrast, *Gldc*^–/^*^–^* littermates were much smaller, with no detectable somites or age-appropriate morphological structures. **(D,E)** A subset of embryos were already significantly smaller than littermates at E7.5 (example in **E**); for comparison the region encompassing extra-embryonic/embryonic tissue is indicated by brackets (epc, ectoplacental cone; extra-embryonic endoderm; En, embryonic endoderm). Scale bar represents 1 mm **(A–C)** or 0.1 mm **(D,E)**.

Additional litters were collected at E8.5 (Table 1A) and among five litters (*n* = 48 embryos), seven implantations (15%) contained tissue masses within the yolk sac that genotyped as *Gcsh*^–^*^/^*^–^, but which had not developed recognizable embryonic features such as head-folds or somites (Figure 2). Small size and abnormal embryonic structures, presumably corresponding to homozygous null embryos, was also noted by E7.5 (Figure 2).

### Heterozygous *Gcsh^+/–^* Mice Do Not Exhibit Elevated Plasma Glycine

A hallmark of loss of function of the GCS in humans and mice is the presence of elevated glycine concentration in tissue and body fluids. Although this could not be assessed in homozygous mutants, we compared plasma glycine concentration in wild-type and *Gcsh*^+/–^ adult mice (*n* = 3 per genotype). The plasma glycine concentration in *Gcsh*^+/–^ (391 ± 72 μM; mean ± *SD*) did not significantly differ from that in wild-type mice (347 ± 16 μM), suggesting that loss of one Gcsh allele did not cause appreciable suppression of glycine cleavage system activity.

### Early Lethality of *Gcsh* Null Embryos Cannot Be Rescued by Formate Supplementation

In addition to regulation of glycine abundance, a key activity of the GCS is the provision of glycine-derived one carbon units to folate one-carbon metabolism (FOCM), with downstream flux into nucleotide biosynthesis and methylation reactions. Suppression of FOCM is implicated in the causation of structural malformations in *Gldc*-deficient embryos, including NTDs and ventriculomegaly (Leung et al., 2017; Santos et al., 2020). Hence, these abnormalities can be rescued by provision of additional one-carbon groups to the folate cycle, for example via maternal supplementation with formate (Pai et al., 2015; Leung et al., 2017; Santos et al., 2020).

In order to test whether formate treatment would enhance development of *Gcsh*^–/–^ embryos, heterozygous timed matings were performed and pregnant *Gcsh*^+/–^ females were supplemented with 30 mg/ml formate in the drinking water from E0.5 as in previous studies (Pai et al., 2015). Among litters collected at E8.5-10.5, the frequency of resorption was similar to that observed among non-supplemented embryos suggesting that lethality had not been prevented (Table 1B). The majority of *Gcsh*^–/–^ embryos among formate-supplemented litters resembled untreated mutants, being considerably smaller than littermates with apparent cessation of morphological development. However, a few *Gcsh*^–/–^ embryos (3 of 10) showed progression to a slightly later stage, with evidence of head-folds and segmentation of 1–2 somites. Nevertheless, at E9.5 these embryos exhibited developmental retardation of least 24–30 h (resembling wild-type embryos at E8.0 or younger) and showed no further developmental progression at E10.5 (Table 1B).

### Heterozygosity for *Gcsh^+/–^* Did Not Increase the Frequency of NTDs Among *Gldc*-Deficient Embryos

Analysis of loss of function models of *Gldc* in mice shows that frequency of NTDs correlates with the degree of suppression of *Gldc* expression, defects occurring among around 25% of *Gldc*^GT1/GT1^ embryos and 60% of *Gldc^–/–^* embryos, which exhibit 90% and 100% reduction in *Gldc* mRNA abundance, respectively (Pai et al., 2015; Leung et al., 2017). The frequency of NTDs is also modified by genetic interaction with a null allele of *Mthfr* (Leung et al., 2017). We therefore tested the hypothesis that the presence of a *Gcsh* null allele may have an additive effect with *Gldc*-deficiency leading to exacerbation of NTDs in *Gldc*^GT1/GT1^ embryos.

The frequency of NTDs was assessed at E10.5 or 13.5 among litters generated by intercross of *Gsch*^+/^*^–^*; *Gldc^GT1/+^* males and *Gldc^GT1/+^* females (Table 2). Surprisingly, we observed NTDs among 67% (*n* = 8 of 12) of *Gldc*^GT1/GT1^ embryos, which is higher than previously observed with this allele of *Gldc* (Pai et al., 2015). It is possible that the penetrance is influenced by alteration of the genetic background, *Gcsh* being on a C57BL/6N background compared with the C57BL/6J background of the *Gldc*^GT1^ line. Nevertheless, the finding that NTDs were present among only 50% of *Gcsh^+/–^*; *Gldc*^GT1/GT1^ embryos compared with 83% of *Gcsh^+/+^*; *Gldc*^GT1/GT1^ embryos (non-significant difference between genotypes) suggests that the introduction of the *Gcsh* null allele did not exacerbate the NTDs in *Gldc*^GT1/GT1^ embryos (Table 2).

**TABLE 2:** Frequency of genotypes and NTDs among litters from *Gcsh****^+/–^***; *Gldc*^GT1/GT1^ × *Gcsh****^+/+^****; Gldc^GT1/GT1^* intercrosses.

		Genotype	*Gcsh*^+/+^			*Gcsh^+/–^*		
			*Gldc*^+/+^	*Gldc^+/GT1^*	*Gldc*^GT1/GT1^	*Gldc*^+/+^	*Gldc^+/GT1^*	*Gldc*^GT1/GT1^
			
	Litters	Embryos	No. embryos (NTDs)			No. embryos (NTDs)		
E10.5	3	16	1 (0)	5 (0)	4 (4)	2 (0)	2 (0)	2 (1)
E13.5	3	20	1 (0)	5 (0)	2 (1)	1 (0)	7 (0)	4 (2)
Total	7	36	2 (0)	10 (0)	6 (5) = 83%	3	9	6 (3) = 50%

**NTDs only arose among Gldc^*GT1/GT1*^ embryos and there was no significant effect of Gcsh genotype (Fisher Exact test).**

## Discussion

Loss of function of Gcsh in mice results in early lethality, prior to E8.5, such that a requirement for Gcsh function in neural tube closure or later brain development and function could not be evaluated. This phenotype of *Gcsh* null embryos differed from that of *Amt* and *Gldc* in which embryos survive to at least perinatal stages, with death at or shortly after birth resulting from NTDs in affected pups (Narisawa et al., 2012; Leung et al., 2017). Overall, these findings indicate that the lethal effect of Gsch mutation at pre-neurulation stages is unlikely to result solely from loss of function of the GCS. However, a few formate supplemented *Gcsh* null embryos appeared to show a slight improvement in development compared with untreated embryos, suggesting that the suppression of FOCM may contribute in part to early lethality. The prevention of NTDs in *Gldc* mutant embryos is associated with increase in cell proliferation, perhaps via enhanced nucleotide biosynthesis, so it is possible that such an effect also supports earlier development in *Gcsh* mutants.

**FIGURE 3:**
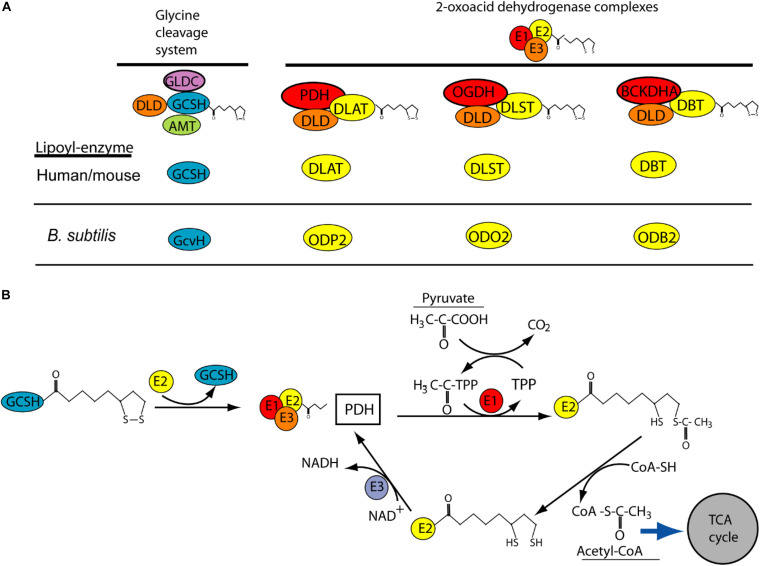
Potential role of GCSH as a donor of lipoic acid to multiple lipoylated-proteins. **(A)** Lipoylation is required for function of GCSH in the glycine cleavage system, and for transfer or lipoyl groups to subunits of 2-oxoacid dehydrogenase complexes including pyruvate dehydrogenase (PDH), 2-oxyglutarate dehydrogenase (OGDH) and branched-chain alpha keto-dehydrogenase (BCKDHA). These complexes are highly conserved (orthologs in *B. subtilis* are shown). **(B)** Possible mechanism by which GSCH acts as lipoyl-donor to the E2 subunit of pyruvate dehydrogenase, whose action leads to production of acetyl-CoA. This activity has been demonstrated in *B. subtilis* but not yet in human or mouse.

The requirement for GCSH function during early development may explain why homozygous or compound heterozygous mutations have not been reported in NKH patients. Similarly, NKH patients have not been found to carry *DLD* mutations, while knockout of *Dld* in mice results in embryonic lethality prior to E9.5, with developmental delay apparent by E7.5 (Johnson et al., 1997). This early lethality of *Dld* null embryos is thought to be due to the requirement for DLD function for multiple enzymes, rather than specifically the GCS. DLD acts as the so called E3 component (as dihydrolipoamide dehydrogenase) of the GCS and three other mitochondrial multienzyme complexes, known as 2-oxoacid dehydrogenase complexes (Figure 3A). These complexes, pyruvate dehydrogenase, α-ketogutarate dehydrogenase and branched-chain α-ketoacid dehydrogenase, are required for aerobic metabolism and branched-chain fatty acid synthesis. In these complexes, DLD or E3 is tightly bound to the E1 and E2 subunits where E1 is a thiamine pyrophosphate-dependent 2-oxoacid dehydrogenase. Interestingly, E2 subunits of the complexes are highly conserved and like GCSH they require covalent attachment of lipoic acid, as a cofactor, to form the active lipoyl-enzyme (Figure 3A).

Like DLD, GCSH may have functions in addition to the requirement in the GCS. This concept is supported by recent studies of the *GCSH* ortholog, *GcvH*, in *B. subtilis* (Cao et al., 2018a). Unlike in other bacteria, such as *E. coli*, where GcvH functions only in the GCS, *B. subtilis* GcvH can act as lipoyl-moiety donor in the biosynthetic modification of other lipoic acid requiring 2-oxoacid dehydrogenase proteins. Moreover, human GCSH can substitute for loss of *GcvH* in *B. subtilis* (Cao et al., 2018a). GSCH may act as a master donor of the lipoyl group to E2 subunit of other 2-oxoacid dehydrogenase complexes such as pyruvate dehydrogenase where its function is crucial for the tricarboxylic acid (TCA) cycle (Figure 3B).

Interestingly, the transcriptional regulation of *GcvH* is also separate from other GCS components in *B. subtilis*, unlike in *E. coli* where it is co-regulated with *GcvP* and *GcvT* in response to glycine (Cao et al., 2018a). Tissue expression of *GCSH* in higher vertebrates may also occur in sites that do not express the specific GCS components (*GLDC* and *AMT*), suggesting a possible requirement beyond that in the GCS. For example, the expression level of *GCSH* in chick correlated with that of *GLDC* in liver, kidney, and brain but mRNA was also detected in other sites including heart, spleen and skeletal muscle where *GLDC* was absent (Kure et al., 1991). In mouse embryos *Gldc* is abundantly expressed throughout the neuroepithelium during neural tube closure (Pai et al., 2015) but becomes more restricted at later stages, with post-natal expression being highest in liver, kidney and brain, whereas *Gcsh* also is expressed in *Gldc*-negative tissue such as skeletal muscle (Gene expression database (GXD)^2^, October 2020). Similarly, human mRNA and protein databases reveal a more widespread tissue distribution of GCSH than GLDC, which is primarily expressed in liver, kidney and brain (Human Protein Atlas)^3^ (Uhlen et al., 2015).

## Conclusion

In conclusion, Gcsh activity is required for embryonic survival in mice. We hypothesize that Gcsh has GCS-independent activity as a lipoate relay that is required for post-implantation development in mice and humans.

## Data Availability Statement

The original contributions presented in the study are included in the article/supplementary material, further inquiries can be directed to the corresponding author/s.

## Ethics Statement

The animal study was reviewed and approved by the University College London Animal Welfare Review Board.

## Author Contributions

NG, K-YL, and AC conceived the study. NG, K-YL, SDC, and GG carried out the experimental work. NG and K-YL drafted the manuscript. All authors analyzed the data and edited the manuscript.

## Conflict of Interest

The authors declare that the research was conducted in the absence of any commercial or financial relationships that could be construed as a potential conflict of interest.

## Footnotes

1 www.mousephenotype.org

2 http://www.informatics.jax.org

3 http://www.proteinatlas.org
